# Supplementary material for: A Multimodal Desorption Electrospray Ionisation Workflow Enabling Visualisation of Lipids and Biologically Relevant Elements in a Single Tissue Section
Source: Metabolites. 2023 Feb 11;13(2):262. doi: 10.3390/metabo13020262 (PMC9964958; doi:10.3390/metabo13020262)
Supplement: Supplementary file 1 [file metabolites-13-00262-s001.zip › metabolites-2210339-supplementary.pdf]

# A multimodal desorption electrospray ionisation workflow enabling visualisation of lipids and biologically relevant elements in a single tissue section

Catia Costa [1], Janella De Jesus [2,3], Chelsea Nikula [3], Teresa Murta [3], Geoffrey W. Grime [1], Vladimir Palitsin [1], Véronique Dartois [4], Kaya Firat [4], Roger Webb [1], Josephine Bunch [3] and Melanie J. Bailey [1,2,*]

[1] University of Surrey Ion Beam Centre, Guildford GU2 7XH, UK; c.d.costa@surrey.ac.uk (C.C.); g.grime@surrey.ac.uk (G.W.G.); v.palitsin@surrey.ac.uk (V.P.); r.webb@surrey.ac.uk (R.W.)
[2] Department of Chemistry, University of Surrey, Guildford GU2 7XH, UK; janella.marie.de.jesus@npl.co.uk
[3] The National Physical Laboratory, Teddington TW11 0LW, UK; cjnikula@gmail.com (C.N.); tigoncalvesmurta@gmail.com (T.M.); josephine.bunch@npl.co.uk (J.B.)
[4] Center for Discovery and Innovation, Hackensack Meridian School of Medicine, Nutley, NJ 07110, USA; veronique.dartois@hmh-cdi.org (V.D.); firat.kaya@hmh-cdi.org (K.F.)
[*] Correspondence: m.bailey@surrey.ac.uk; Tel.: +44-(0)1483682593

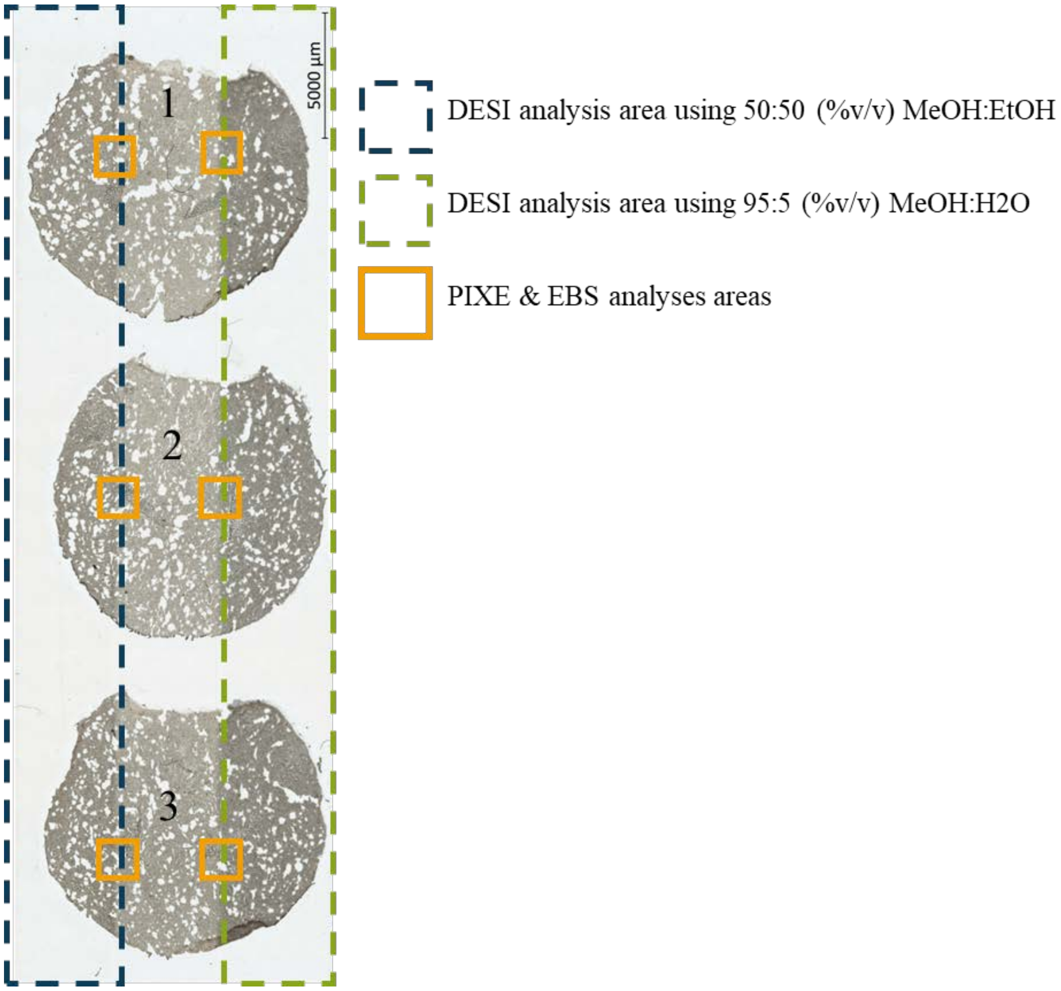


**Figure S1.** Areas analysed by DESI, PIXE & EBS on liver tissue homogenate sections.

## (A)

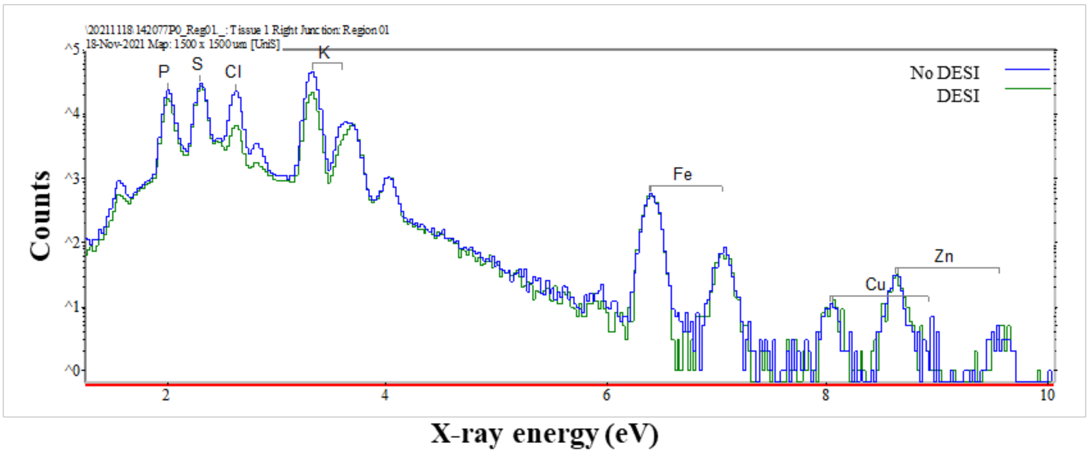


## (B)

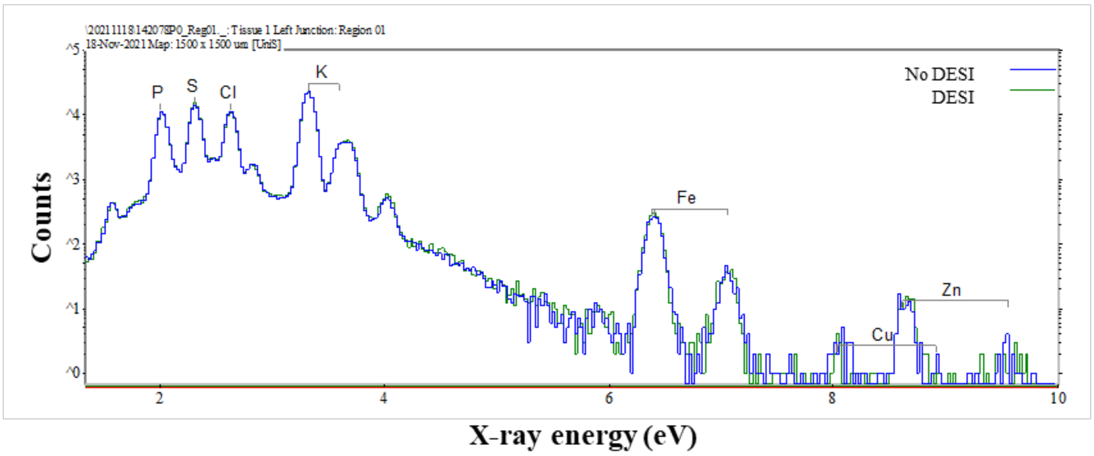


**Figure S2.** Overlay of X-ray spectra taken from regions of interest on areas not analysed (Reg01; blue) and analysed by DESI (Reg02; green) using (A) 95:5 (%v/v) MeOH:$H_2O$ and (B) 50:50 (%v/v) MeOH:EtOH.

**Table S1.** T-test results between the average (n=3) of the ratios of the elemental peak area measured by PIXE after DESI analysis with two different solvents – MeOH/$H_2O$ and MeOH/EtOH.

| Table Analyzed | P |
|---|---|
| | |
| Column B | MeOH/EtOH |
| vs. | vs. |
| Column A | MeOH/H2O |
| | |
| Unpaired t test | |
| P value | 0.0004 |
| P value summary | *** |
| Significantly different (P < 0.05)? | Yes |
| One- or two-tailed P value? | Two-tailed |
| t, df | t=11.28, df=4 |
| | |
| How big is the difference? | |
| Mean of column A | 0.6233 |
| Mean of column B | 1.007 |
| Difference between means (B - A) ± SEM | 0.3833 ± 0.03399 |
| 95% confidence interval | 0.2890 to 0.4777 |
| R squared (eta squared) | 0.9695 |
| | |
| F test to compare variances | |
| F, DFn, Dfd | 13.86, 2, 2 |
| P value | 0.1346 |
| P value summary | ns |
| Significantly different (P < 0.05)? | No |
| | |
| Data analyzed | |
| Sample size, column A | 3 |
| Sample size, column B | 3 |

| Table Analyzed | S |
|---|---|
| | |
| Column B | MeOH/EtOH |
| vs. | vs. |
| Column A | MeOH/H2O |
| | |
| Unpaired t test | |
| P value | 0.0016 |
| P value summary | ** |
| Significantly different (P < 0.05)? | Yes |
| One- or two-tailed P value? | Two-tailed |
| t, df | t=7.566, df=4 |
| | |
| How big is the difference? | |
| Mean of column A | 0.7667 |
| Mean of column B | 1.087 |
| Difference between means (B - A) ± SEM | 0.3200 ± 0.04230 |
| 95% confidence interval | 0.2026 to 0.4374 |
| R squared (eta squared) | 0.9347 |
| | |
| F test to compare variances | |
| F, DFn, Dfd | 39.25, 2, 2 |
| P value | 0.0497 |
| P value summary | * |
| Significantly different (P < 0.05)? | Yes |
| | |
| Data analyzed | |
| Sample size, column A | 3 |
| Sample size, column B | 3 |

| Table Analyzed | Cl |
|---|---|
| | |
| Column B | MeOH/EtOH |
| vs. | vs. |
| Column A | MeOH/H2O |
| | |
| Unpaired t test | |
| P value | <0.0001 |
| P value summary | **** |
| Significantly different (P < 0.05)? | Yes |
| One- or two-tailed P value? | Two-tailed |
| t, df | t=25.22, df=4 |
| | |
| How big is the difference? | |
| Mean of column A | 0.2567 |
| Mean of column B | 0.96 |
| Difference between means (B - A) ± SEM | 0.7033 ± 0.02789 |
| 95% confidence interval | 0.6259 to 0.7808 |
| R squared (eta squared) | 0.9938 |
| | |
| F test to compare variances | |
| F, DFn, Dfd | 9.000, 2, 2 |
| P value | 0.2 |
| P value summary | ns |
| Significantly different (P < 0.05)? | No |
| | |
| Data analyzed | |
| Sample size, column A | 3 |
| Sample size, column B | 3 |

| Table Analyzed | K |
|---|---|
| | |
| Column B | MeOH/EtOH |
| vs. | vs. |
| Column A | MeOH/H2O |
| | |
| Unpaired t test | |
| P value | <0.0001 |
| P value summary | **** |
| Significantly different (P < 0.05)? | Yes |
| One- or two-tailed P value? | Two-tailed |
| t, df | t=21.57, df=4 |
| | |
| How big is the difference? | |
| Mean of column A | 0.4 |
| Mean of column B | 1.027 |
| Difference between means (B - A) ± SEM | 0.6267 ± 0.02906 |
| 95% confidence interval | 0.5460 to 0.7073 |
| R squared (eta squared) | 0.9915 |
| | |
| F test to compare variances | |
| F, DFn, Dfd | 7.444, 2, 2 |
| P value | 0.2368 |
| P value summary | ns |
| Significantly different (P < 0.05)? | No |
| | |
| Data analyzed | |
| Sample size, column A | 3 |
| Sample size, column B | 3 |

| Table Analyzed | Fe |
|---|---|
| | |
| Column B | MeOH/EtOH |
| vs. | vs. |
| Column A | MeOH/H2O |
| | |
| Unpaired t test | |
| P value | 0.0067 |
| P value summary | ** |
| Significantly different (P < 0.05)? | Yes |
| One- or two-tailed P value? | Two-tailed |
| t, df | t=5.163, df=4 |
| | |
| How big is the difference? | |
| Mean of column A | 0.8067 |
| Mean of column B | 1.09 |
| Difference between means (B - A) ± SEM | 0.2833 ± 0.05487 |
| 95% confidence interval | 0.1310 to 0.4357 |
| R squared (eta squared) | 0.8695 |
| | |
| F test to compare variances | |
| F, DFn, Dfd | 2.430, 2, 2 |
| P value | 0.583 |
| P value summary | ns |
| Significantly different (P < 0.05)? | No |
| | |
| Data analyzed | |
| Sample size, column A | 3 |
| Sample size, column B | 3 |

| Table Analyzed | Zn |
|---|---|
| | |
| Column B | MeOH/EtOH |
| vs. | vs. |
| Column A | MeOH/H2O |
| | |
| Unpaired t test | |
| P value | 0.0232 |
| P value summary | * |
| Significantly different (P < 0.05)? | Yes |
| One- or two-tailed P value? | Two-tailed |
| t, df | t=3.577, df=4 |
| | |
| How big is the difference? | |
| Mean of column A | 0.87 |
| Mean of column B | 1.107 |
| Difference between means (B - A) ± SEM | 0.2367 ± 0.06616 |
| 95% confidence interval | 0.05296 to 0.4204 |
| R squared (eta squared) | 0.7618 |
| | |
| F test to compare variances | |
| F, DFn, Dfd | 5.254, 2, 2 |
| P value | 0.3198 |
| P value summary | ns |
| Significantly different (P < 0.05)? | No |
| | |
| Data analyzed | |
| Sample size, column A | 3 |
| Sample size, column B | 3 |

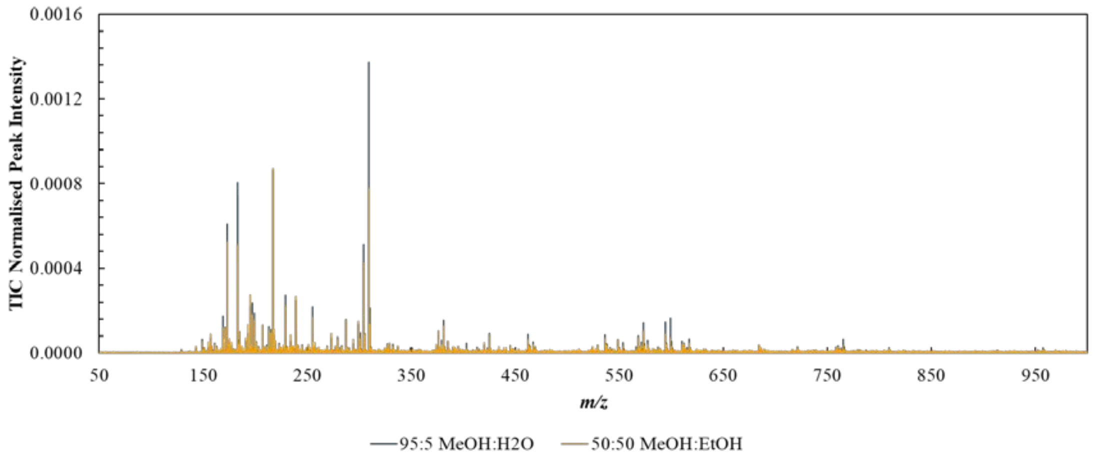


**Figure S3.** Spectra acquired with DESI using the two solvent systems 95:5 (%v/v) methanol/water (top) or 50:50 (%v/v) methanol/ethanol from the background (PET-only).

**Table S2.** Top 50 lipid features, adducts formed and measured *m/z* detected using each spray solvent system.

| 95:5 (%v/v) MeOH:$H_2O$ | | |
|---|---|---|
| **Assignment** | **Adduct** | **Measured m/z** |
| **PC 36:4** | $[M+K]^+$ | 820.538 |
| **PC 38:4** | $[M+K]^+$ | 848.568 |
| **PC 34:2** | $[M+K]^+$ | 796.534 |
| **PC 38:7** | $[M+H]^+$ | 804.562 |
| **PC 40:7** | $[M+H]^+$ | 832.592 |
| **PC 36:5** | $[M+H]^+$ | 780.560 |
| **PC 34:1** | $[M+K]^+$ | 798.550 |
| **PC 36:4** | $[M+H]^+$ | 782.574 |
| **PC 36:2** | $[M+K]^+$ | 824.566 |
| **PA 42:3** | $[M+K]^+$ | 821.542 |
| **PG 42:7** | $[M+H]^+$ | 849.570 |
| **PC 38:5** | $[M+H]^+$ | 808.592 |
| **PC 36:3** | $[M+K]^+$ | 822.546 |
| **PA 40:1** | $[M+K]^+$ | 797.540 |
| **PC 38:5** | $[M+K]^+$ | 846.548 |
| **FA 18:2** | $[M+K]^+$ | 319.226 |
| **PC 38:6** | $[M+H]^+$ | 806.572 |
| **PC 38:6** | $[M+K]^+$ | 844.536 |
| **PC 38:4** | $[M+H]^+$ | 810.606 |
| **PA 42:1** | $[M+K]^+$ | 825.572 |
| **PA 40:0** | $[M+Na]^+$ | 783.582 |
| **PC 38:3** | $[M+K]^+$ | 850.574 |
| **PG 38:4** | $[M+H]^+$ | 799.554 |
| **PC 38:5** | $[M+Na]^+$ | 830.578 |
| **PC 38:6** | $[M+Na]^+$ | 828.552 |
| **LPC 16:0** | $[M+K]^+$ | 534.302 |
| **FA 20:5** | $[M+H_3O]^+$ | 321.244 |
| **PC 36:1** | $[M+K]^+$ | 826.572 |
| **PC 34:2** | $[M+H]^+$ | 758.578 |
| **PG 40:5** | $[M+Na]^+$ | 847.556 |
| **PC 40:6** | $[M+H]^+$ | 834.606 |
| **PG 40:6** | $[M+H]^+$ | 823.550 |
| **LPC 18:3** | [M+H]+ | 518.328 |
| **PC 40:6** | $[M+K]^+$ | 872.564 |
| **PC 32:1** | $[M+K]^+$ | 770.516 |
| **LPC 20:4** | $[M+K]^+$ | 582.302 |
| **PG 40:6** | $[M+Na]^+$ | 845.536 |
| **PS 36:1** | $[M+Na]^+$ | 812.542 |

| 50:50 (%v/v) MeOH:EtOH | | |
|---|---|---|
| **Assignment** | **Adduct** | **Measured m/z** |
| **PC 36:4** | $[M+K]^+$ | 820.538 |
| **PC 38:4** | $[M^+K]^+$ | 848.568 |
| **PC 38:7** | $[M^+H]^+$ | 804.562 |
| **PC 40:7** | $[M^+H]^+$ | 832.596 |
| **PC 34:2** | $[M+K]^+$ | 796.54 |
| **PC 36:4** | $[M^+H]^+$ | 782.58 |
| **PA 42:3** | $[M^+K]^+$ | 821.542 |
| **PC 34:1** | $[M^+K]^+$ | 798.552 |
| **PI 32:0** | $[M^+K]^+$ | 849.576 |
| **PC 36:2** | $[M+K]^+$ | 824.572 |
| **PC 38:5** | $[M^+H]^+$ | 808.596 |
| **PC 36:3** | $[M^+K]^+$ | 822.55 |
| **PA 40:1** | $[M^+K]^+$ | 797.544 |
| **PC 38:5** | $[M+K]^+$ | 846.554 |
| **PC 38:6** | $[M^+H]^+$ | 806.578 |
| **PC 38:4** | $[M^+H]^+$ | 810.612 |
| **PC 38:5** | $[M^+Na]^+$ | 830.578 |
| **PC 38:6** | $[M^+K]^+$ | 844.536 |
| **PC 38:3** | $[M^+K]^+$ | 850.578 |
| **PA 40:0** | $[M^+Na]^+$ | 783.582 |
| **PC 40:6** | $[M^+H]^+$ | 834.606 |
| **PA 42:1** | $[M^+K]^+$ | 825.572 |
| **PG 38:4** | $[M+H]^+$ | 799.558 |
| **PG 40:5** | $[M+Na]^+$ | 847.56 |
| **PC 36:1** | $[M^+K]^+$ | 826.578 |
| **FA 18:2** | $[M+K]^+$ | 319.23 |
| **PG 40:6** | $[M^+H]^+$ | 823.55 |
| **PG 40:6** | $[M^+Na]^+$ | 845.542 |
| **PA 42:0** | $[M^+Na]^+$ | 811.616 |
| **LPC 18:3** | $[M^+H]^+$ | 518.33 |
| **PC 36:3** | $[M^+H]^+$ | 784.592 |
| **PC32:1** | $[M+K]^+$ | 770.522 |
| **PC 36:2** | $[M^+H]^+$ | 786.612 |
| **PE O-40:6** | $[M^+Na]^+$ | 800.556 |
| **PC 34:1** | $[M^+H]^+$ | 760.592 |
| **FA 20:5** | $[M^+H]^+$ | 303.234 |
| **LPC 20:4** | $[M^+Na]^+$ | 566.33 |
| **LPC 20:3** | $[M^+H]^+$ | 546.362 |

| | | |
|---|---|---|
| **LPC 18:0** | [M+K]$^+$ | 562.334 |
| **PC 42:11** | [M+H]$^+$ | 852.538 |
| **PA 42:0** | [M+Na]$^+$ | 811.610 |
| **PC 40:6** | [M+Na]$^+$ | 856.584 |
| **PS 38:3** | [M+Na]$^+$ | 836.536 |
| **LPC 20:4** | [M+Na]$^+$ | 566.330 |
| **PC 36:3** | [M+H]$^+$ | 784.590 |
| **PC 42:8** | [M+K]$^+$ | 896.558 |
| **PE O-40:6** | [M+Na]$^+$ | 800.554 |
| **PC 34:1** | [M+H]$^+$ | 760.592 |
| **LPC 20:3** | [M+H]$^+$ | 546.360 |
| **PC 36:2** | [M+H]$^+$ | 786.610 |

| | | |
|---|---|---|
| **PC 34:5** | [M+K]$^+$ | 790.548 |
| **PG 42:6** | [M$^+$H]$^+$ | 851.582 |
| **LPC 20:4** | [M$^+$K]$^+$ | 582.304 |
| **FA 18:1** | [M+K]$^+$ | 321.244 |
| **LPC 18:0** | [M$^+$K]$^+$ | 562.334 |
| **PI 34:2** | [M+K]$^+$ | 873.574 |
| **PE 38:2** | [M$^+$H]$^+$ | 772.538 |
| **PC 40:5** | [M$^+$K]$^+$ | 874.58 |
| **FA 22:6** | [M$^+$K]$^+$ | 367.204 |
| **FA 20:4** | [M$^+$Na]$^+$ | 327.234 |
| **FA 18:2** | [M$^+$K]$^+$ | 319.208 |
| **PE 38:1** | [M$^+$K]$^+$ | 812.548 |

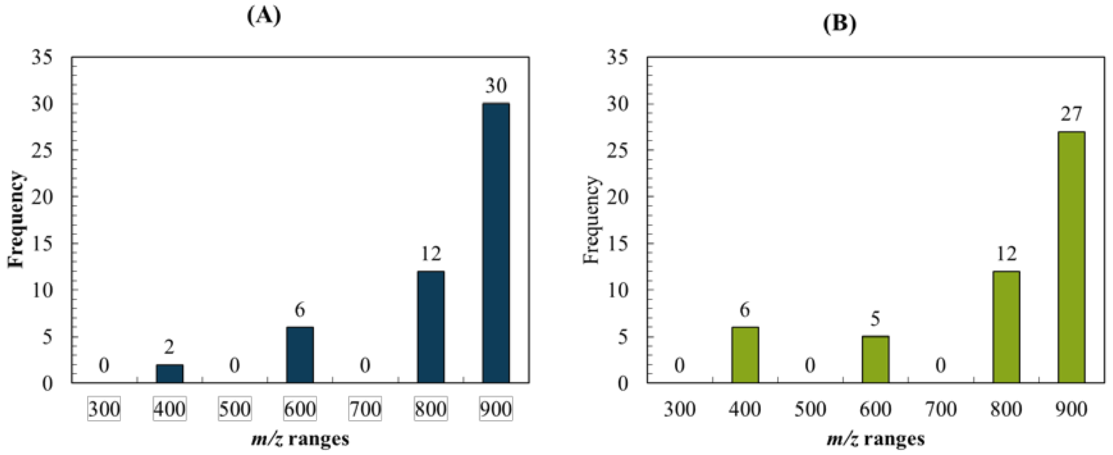


**Figure S4.** Histogram of the number of lipid features in the top 50 detected per m/z range (bin size 100) for each spray solvent – (A) MeOH/$H_2O$ and (B) MeOH/EtOH.

**Table S3.** T-test results comparing the measured normalised (to TIC) peak intensities for the top 10 lipids measured using the two solvent systems.

| | Discovery? | P value | Mean of 95:5 (%v/v) MeOH:H2O | Mean of 50:50 (%v/v/) MeOH:EtOH | Difference | SE of difference | t ratio | df | q value |
|---|---|---|---|---|---|---|---|---|---|
| **PC 36:4 $[M+K]^+$** | Yes | <0.000001 | 0.01117 | 0.01398 | -0.002816 | 0.0003404 | 8.27 | 16 | <0.000001 |
| **PC 38:4 $[M+K]^+$** | Yes | <0.000001 | 0.01014 | 0.01349 | -0.003351 | 0.0003131 | 10.7 | 16 | <0.000001 |
| **PC 34:2 $[M+K]^+$** | Yes | 0.000786 | 0.009648 | 0.01054 | -0.0008878 | 0.000215 | 4.13 | 16 | 0.000893 |
| **PC 38:7 $[M+H]^+$** | Yes | <0.000001 | 0.007512 | 0.01219 | -0.004677 | 0.0002612 | 17.91 | 16 | <0.000001 |
| **PC 40:7 $[M+H]^+$** | Yes | <0.000001 | 0.006606 | 0.0115 | -0.004899 | 0.0002348 | 20.86 | 16 | <0.000001 |
| **PC 34:1 $[M+K]^+$** | Yes | 0.006748 | 0.007 | 0.007458 | -0.0004578 | 0.0001472 | 3.109 | 16 | 0.006816 |
| **PC 36:4 $[M+H]^+$** | Yes | <0.000001 | 0.006516 | 0.008541 | -0.002026 | 0.0001099 | 18.44 | 16 | <0.000001 |
| **PC 36:2 $[M+K]^+$** | Yes | 0.000004 | 0.006268 | 0.007224 | -0.0009567 | 0.000139 | 6.884 | 16 | 0.000005 |
| **PA 42:3 $[M+K]^+$** | Yes | 0.000001 | 0.00551 | 0.006727 | -0.001217 | 0.0001635 | 7.439 | 16 | 0.000002 |

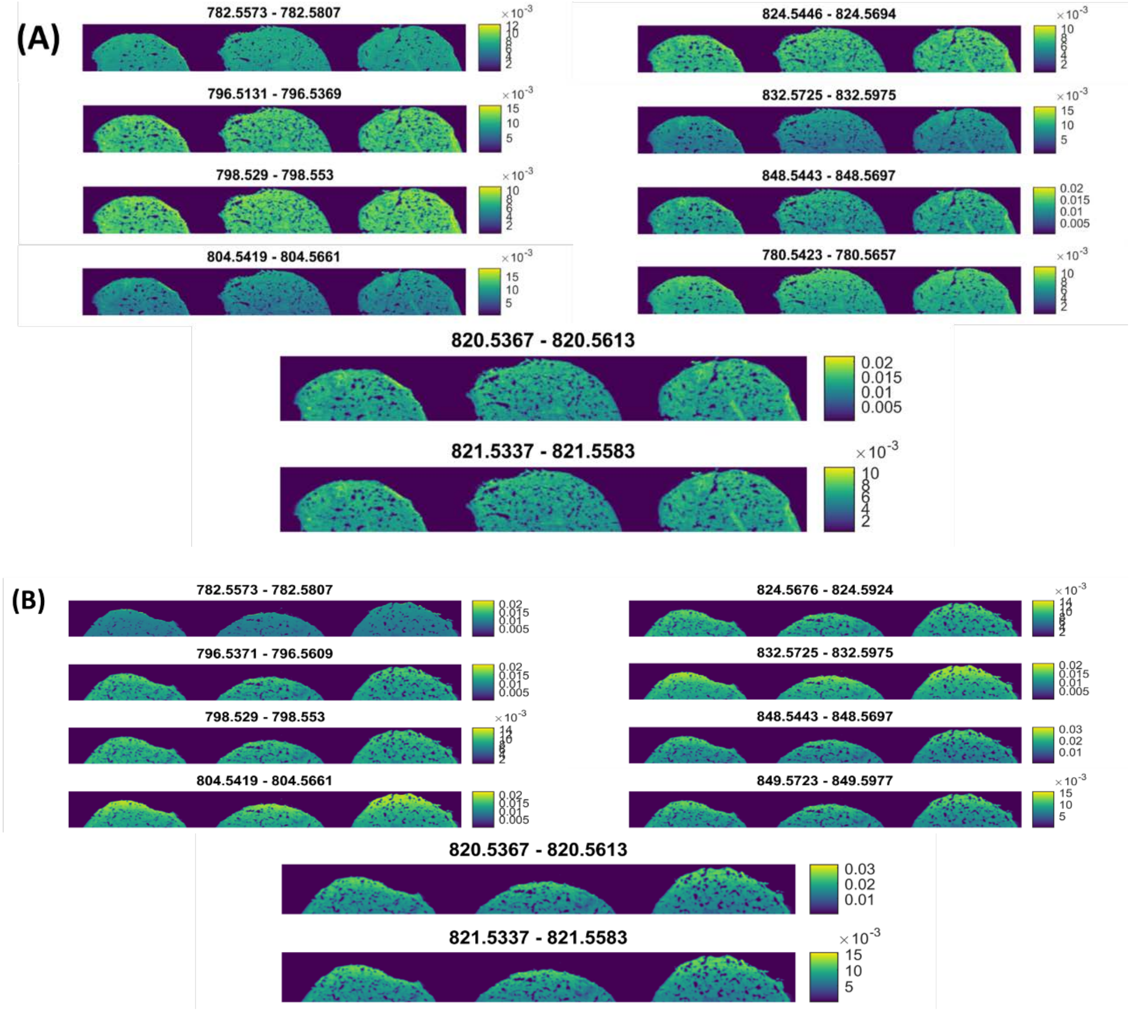


**Figure S5.** Resulting DESI ion maps for the top 10 most abundant lipids imaged using (A) 95:5 (%v/v) MeOH:$H_2O$ or (B) 50:50 (%v/v) MeOH:EtOH spray solvents on liver tissue homogenates.

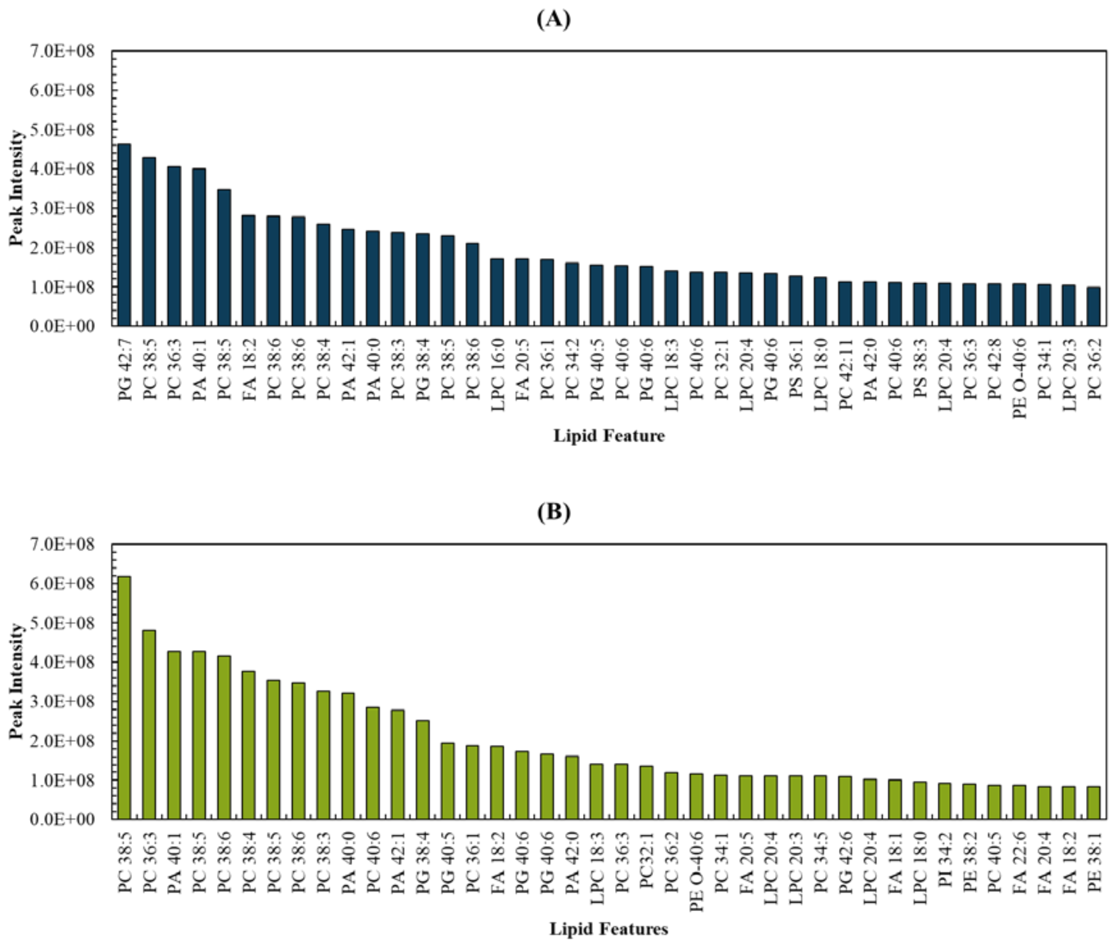


**Figure S6.** Remaining 40 features in the top 50 most intense lipid features and their respective intensities for (A) MeOH:H2O and (B) MeOH:EtOH.

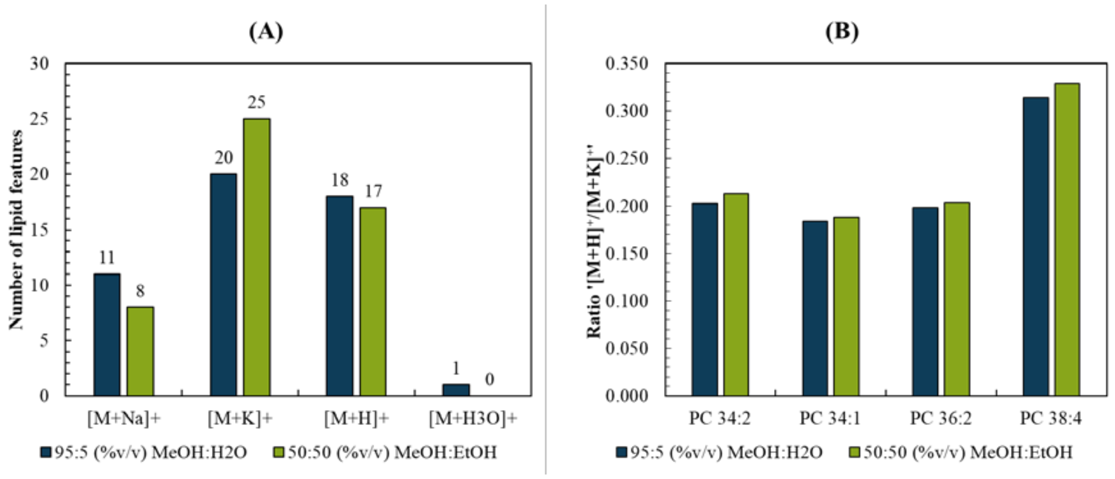


**Figure S7.** (A) Number of lipid features detected in the top 50 lipids with a specific adduct for each of the two solvent mixtures – 95:5 (%v/v) MeOH:$H_2O$ (blue) and 50:50 (%v/v) MeOH:EtOH (green). (B) Ratio of average (n=9) peak intensities measured for $[M+H]^+$ and $[M+K]^+$ ions formed for a selection of PC lipids using the two solvent mixtures – 95:5 (%v/v) MeOH:$H_2O$ (blue) and 50:50 (%v/v) MeOH:EtOH (green).

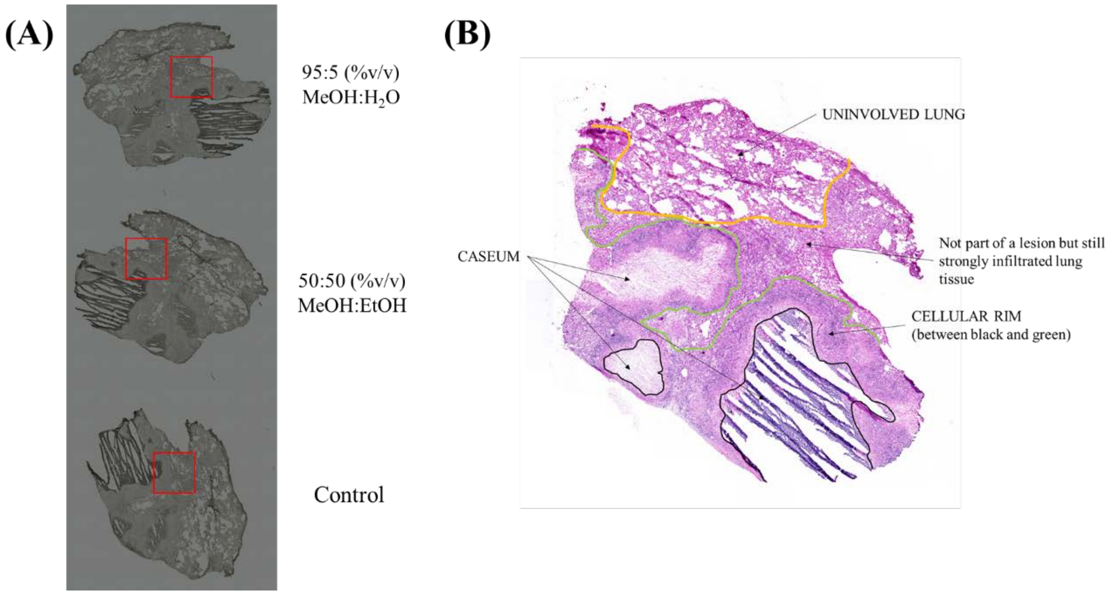


**Figure S8.** (A) Optical images of sequential fresh frozen rabbit lung tissues containing a caseous granuloma (a lesion caused by tuberculosis) showing the regions analysed using ion beam analysis; (B) Hematoxylin and eosin (H&E) stained section and the different regions of the granuloma.

**Table S4.** T-test results of the elemental peak area measured by PIXE from a lung tissue section after DESI analysis with two different solvents – MeOH/$H_2O$ and MeOH/EtOH.

| Table Analyzed | Phosphorus |
|---|---|
| Column B | MeOH:H2O |
| vs. | vs. |
| Column A | Control |
| Unpaired t test | |
| P value | 0.0042 |
| P value summary | ** |
| Significantly different (P < 0.05)? | Yes |
| One- or two-tailed P value? | Two-tailed |
| t, df | t=4.484, df=6 |
| How big is the difference? | |
| Mean of column A | 169825 |
| Mean of column B | 103629 |
| Difference between means (B - A) ± SEM | -66197 ± 14763 |
| 95% confidence interval | -102321 to -30073 |
| R squared (eta squared) | 0.7702 |
| F test to compare variances | |
| F, DFn, Dfd | 1.472, 3, 3 |
| P value | 0.7585 |
| P value summary | ns |
| Significantly different (P < 0.05)? | No |
| Data analyzed | |
| Sample size, column A | 4 |
| Sample size, column B | 4 |

| Table Analyzed | Sulphur |
|---|---|
| Column B | MeOH:H2O |
| vs. | vs. |
| Column A | Control |
| Unpaired t test | |
| P value | 0.0641 |
| P value summary | ns |
| Significantly different (P < 0.05)? | No |
| One- or two-tailed P value? | Two-tailed |
| t, df | t=2.265, df=6 |
| How big is the difference? | |
| Mean of column A | 166966 |
| Mean of column B | 131636 |
| Difference between means (B - A) ± SEM | -35330 ± 15598 |
| 95% confidence interval | -73496 to 2836 |
| R squared (eta squared) | 0.4609 |
| F test to compare variances | |
| F, DFn, Dfd | 2.191, 3, 3 |
| P value | 0.536 |
| P value summary | ns |
| Significantly different (P < 0.05)? | No |
| Data analyzed | |
| Sample size, column A | 4 |
| Sample size, column B | 4 |

| Table Analyzed | Chlorine |
|---|---|
| Column B | MeOH:H2O |
| vs. | vs. |
| Column A | Control |
| Unpaired t test | |
| P value | <0.0001 |
| P value summary | **** |
| Significantly different (P < 0.05)? | Yes |
| One- or two-tailed P value? | Two-tailed |
| t, df | t=9.781, df=6 |
| How big is the difference? | |
| Mean of column A | 239292 |
| Mean of column B | 20466 |
| Difference between means (B - A) ± SEM | -218826 ± 22373 |
| 95% confidence interval | -273572 to -164081 |
| R squared (eta squared) | 0.941 |
| F test to compare variances | |
| F, DFn, Dfd | 737.9, 3, 3 |
| P value | 0.0002 |
| P value summary | *** |
| Significantly different (P < 0.05)? | Yes |
| Data analyzed | |
| Sample size, column A | 4 |
| Sample size, column B | 4 |

| Table Analyzed | Potassium |
|---|---|
| Column B | MeOH:H2O |
| vs. | vs. |
| Column A | Control |
| Unpaired t test | |
| P value | <0.0001 |
| P value summary | **** |
| Significantly different (P < 0.05)? | Yes |
| One- or two-tailed P value? | Two-tailed |
| t, df | t=11.11, df=6 |
| How big is the difference? | |
| Mean of column A | 327173 |
| Mean of column B | 90625 |
| Difference between means (B - A) ± SEM | -236548 ± 21288 |
| 95% confidence interval | -288637 to -184459 |
| R squared (eta squared) | 0.9537 |
| F test to compare variances | |
| F, DFn, Dfd | 7.637, 3, 3 |
| P value | 0.129 |
| P value summary | ns |
| Significantly different (P < 0.05)? | No |
| Data analyzed | |
| Sample size, column A | 4 |
| Sample size, column B | 4 |

| Table Analyzed | Iron |
|---|---|
| Column B | MeOH:H2O |
| vs. | vs. |
| Column A | Control |
| Unpaired t test | |
| P value | 0.354 |
| P value summary | ns |
| Significantly different (P < 0.05)? | No |
| One- or two-tailed P value? | Two-tailed |
| t, df | t=1.004, df=6 |
| How big is the difference? | |
| Mean of column A | 2756 |
| Mean of column B | 2233 |
| Difference between means (B - A) ± SEM | -523.1 ± 520.8 |
| 95% confidence interval | -1797 to 751.2 |
| R squared (eta squared) | 0.1439 |
| F test to compare variances | |
| F, DFn, Dfd | 13.91, 3, 3 |
| P value | 0.0578 |
| P value summary | ns |
| Significantly different (P < 0.05)? | No |
| Data analyzed | |
| Sample size, column A | 4 |
| Sample size, column B | 4 |

| Table Analyzed | Phosphorus |
|---|---|
| Column C | MeOH:EtOH |
| vs. | vs. |
| Column A | Control |
| Unpaired t test | |
| P value | 0.0556 |
| P value summary | ns |
| Significantly different (P < 0.05)? | No |
| One- or two-tailed P value? | Two-tailed |
| t, df | t=2.369, df=6 |
| How big is the difference? | |
| Mean of column A | 169825 |
| Mean of column C | 121313 |
| Difference between means (C - A) ± SEM | -48512 ± 20475 |
| 95% confidence interval | -98613 to 1588 |
| R squared (eta squared) | 0.4834 |
| F test to compare variances | |
| F, DFn, Dfd | 2.231, 3, 3 |
| P value | 0.527 |
| P value summary | ns |
| Significantly different (P < 0.05)? | No |
| Data analyzed | |
| Sample size, column A | 4 |
| Sample size, column C | 4 |

| Table Analyzed | Sulphur |
|---|---|
| Column C | MeOH:EtOH |
| vs. | vs. |
| Column A | Control |
| Unpaired t test | |
| P value | 0.3721 |
| P value summary | ns |
| Significantly different (P < 0.05)? | No |
| One- or two-tailed P value? | Two-tailed |
| t, df | t=0.9643, df=6 |
| How big is the difference? | |
| Mean of column A | 166966 |
| Mean of column C | 146604 |
| Difference between means (C - A) ± SEM | -20362 ± 21115 |
| 95% confidence interval | -72029 to 31306 |
| R squared (eta squared) | 0.1342 |
| F test to compare variances | |
| F, DFn, Dfd | 4.848, 3, 3 |
| P value | 0.2273 |
| P value summary | ns |
| Significantly different (P < 0.05)? | No |
| Data analyzed | |
| Sample size, column A | 4 |
| Sample size, column C | 4 |

| Table Analyzed | Chlorine |
|---|---|
| Column C | MeOH:EtOH |
| vs. | vs. |
| Column A | Control |
| Unpaired t test | |
| P value | 0.2547 |
| P value summary | ns |
| Significantly different (P < 0.05)? | No |
| One- or two-tailed P value? | Two-tailed |
| t, df | t=1.259, df=6 |
| How big is the difference? | |
| Mean of column A | 239292 |
| Mean of column C | 195866 |
| Difference between means (C - A) ± SEM | -43427 ± 34487 |
| 95% confidence interval | -127814 to 40961 |
| R squared (eta squared) | 0.209 |
| F test to compare variances | |
| F, DFn, Dfd | 1.379, 3, 3 |
| P value | 0.7979 |
| P value summary | ns |
| Significantly different (P < 0.05)? | No |
| Data analyzed | |
| Sample size, column A | 4 |
| Sample size, column C | 4 |

| Table Analyzed | Potassium |
|---|---|
| Column C | MeOH:EtOH |
| vs. | vs. |
| Column A | Control |
| Unpaired t test | |
| P value | 0.1484 |
| P value summary | ns |
| Significantly different (P < 0.05)? | No |
| One- or two-tailed P value? | Two-tailed |
| t, df | t=1.658, df=6 |
| How big is the difference? | |
| Mean of column A | 327173 |
| Mean of column C | 259388 |
| Difference between means (C - A) ± SEM | -67785 ± 40881 |
| 95% confidence interval | -167818 to 32248 |
| R squared (eta squared) | 0.3142 |
| F test to compare variances | |
| F, DFn, Dfd | 3.171, 3, 3 |
| P value | 0.3686 |
| P value summary | ns |
| Significantly different (P < 0.05)? | No |
| Data analyzed | |
| Sample size, column A | 4 |
| Sample size, column C | 4 |

| Table Analyzed | Iron |
|---|---|
| Column C | MeOH:EtOH |
| vs. | vs. |
| Column A | Control |
| Unpaired t test | |
| P value | 0.3264 |
| P value summary | ns |
| Significantly different (P < 0.05)? | No |
| One- or two-tailed P value? | Two-tailed |
| t, df | t=1.068, df=6 |
| How big is the difference? | |
| Mean of column A | 2756 |
| Mean of column C | 2127 |
| Difference between means (C - A) ± SEM | -629.8 ± 589.4 |
| 95% confidence interval | -2072 to 812.5 |
| R squared (eta squared) | 0.1599 |
| F test to compare variances | |
| F, DFn, Dfd | 2.680, 3, 3 |
| P value | 0.4396 |
| P value summary | ns |
| Significantly different (P < 0.05)? | No |
| Data analyzed | |
| Sample size, column A | 4 |
| Sample size, column C | 4 |

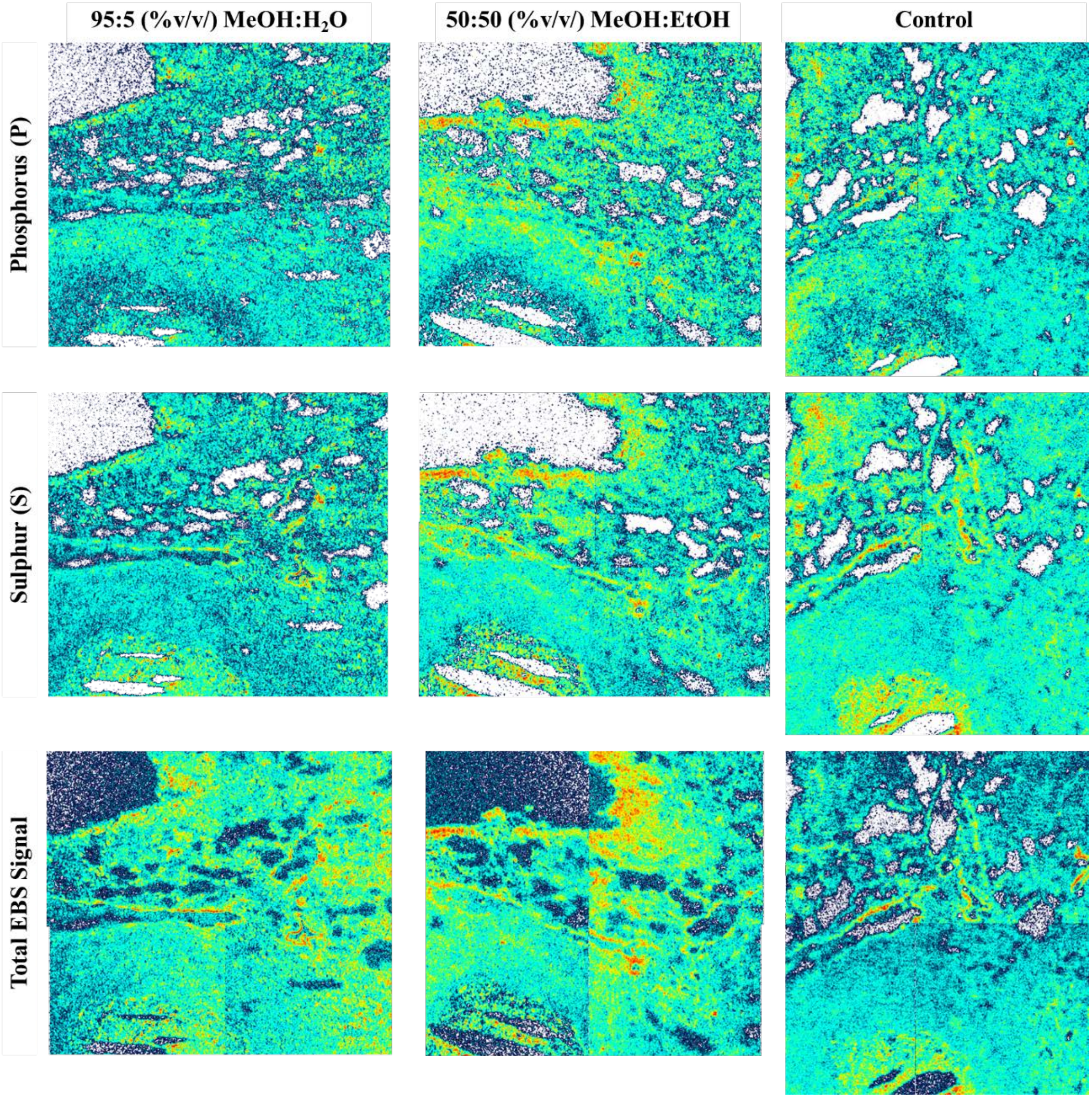


**Figure S9.** Phosphorus (P), sulphur (S) PIXE maps and total EBS maps taken from fresh frozen lung tissue sections after DESI analysis using 95:5 (%v/v) MeOH:H2O or 50:50 (%v/v) MeOH:EtOH. A third section (Control) was also analysed – no DESI measurements were taken on this sample.

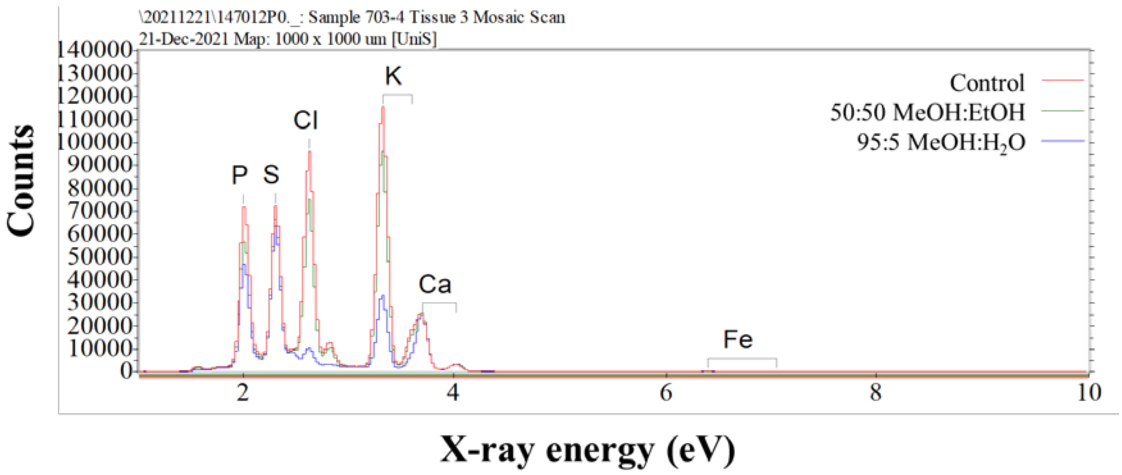


**Figure S10.** Overlay of the X-ray spectra taken from the squares containing the caseum in each of the lung tissue section samples.
